# The reciprocal relationship between material factors and health in the life course: evidence from SHARE and ELSA

**DOI:** 10.1007/s10433-018-0458-3

**Published:** 2018-02-19

**Authors:** Rasmus Hoffmann, Hannes Kröger, Eduwin Pakpahan

**Affiliations:** 1 European University Institute, Fiesole, Italy; 2 Max Planck Institute for Demographic Research, Rostock, Germany; 3 Socio-Economic Panel Study (SOEP), Deutsches Institut für Wirtschaftsforschung e.V. (DIW Berlin), Mohrenstraße 58, 10117 Berlin, Germany

**Keywords:** Material wealth, Health inequality, Life course, Structural equation model

## Abstract

The widely established health differences between people with greater economic resources and those with fewer resources can be attributed to both social causation (material factors affecting health) and health selection (health affecting material wealth). Each of these pathways may have different intensities at different ages, because the sensitivity of health to a lack of material wealth and the degree to which health can influence economic resources may change. We study the relative importance, in terms of explanatory power, of social causation and health selection, comparing the transitions from childhood to adulthood and from adulthood to old age. We use retrospective survey data from ten European countries from the Survey of Health, Ageing and Retirement in Europe (SHARELIFE, *n* = 18,734) and the English Longitudinal Study of Ageing (ELSA, *n* = 6117), and structural equations models in a cross-lagged panel design. Material wealth and health depend on their prior status, wealth more so than health. In the transition from childhood to adulthood, social causation and health selection are equally important: the standardized coefficients for men in SHARE are 0.07 and 0.06, respectively, i.e. one standard deviation increase in material wealth in childhood is associated with a 0.07 standard deviation increase in adult health. In the transition from adulthood to old age, social causation is more important than health selection (0.52 vs. 0.01), across gender and data sets. Both pathways contribute to the creation of health inequalities—however, their relative importance changes with age, which is important for understanding how health inequalities develop and how policies can address them.

**Electronic supplementary material:**

The online version of this article (10.1007/s10433-018-0458-3) contains supplementary material, which is available to authorized users.

## Introduction

Health inequalities between social groups have been found in all periods and countries for which data are available. Morbidity and mortality are systematically higher among people with lower socio-economic status (SES), measured, for example, by education, occupational status, wealth, or income. Health inequalities usually amount to between 5 and 10 years’ difference in life expectancy and between 10 and 20 years’ difference in disability-free life expectancy (Mackenbach 2006), and they rate high on the political agenda (Elo 2009).

Health inequalities in old age are mostly smaller than in adulthood, which has been attributed to a large extent to mortality selection (Crimmins et al. 2009; Hoffmann 2011a), but inequalities between income groups, in particular, are still substantial (Hoffmann 2011b; Huisman et al. 2003). For example, the difference in life expectancy at age 65 in Germany between the lowest and highest pension income quintile is 3.6 years (Shkolnikov et al. 2008). Since the proportion of older people in societies is rapidly increasing, health inequalities among older people are of increasing importance for research and policy. Also increasing is the awareness that health inequalities in old age can be best explained by a life course approach (Mayer 2009), because they are the outcomes of the interaction of two life-long processes: first, the development and changes in a person’s SES, a process with critical periods and transitions, with path dependencies and accumulation; second, health trajectories, determined by fixed individual characteristics, social influences, behaviour, and institutional settings, also with critical periods, path dependencies, and accumulations of health problems or recovery.

The social factors that contribute to health differences can be grouped into material living conditions (e.g. income or wealth), psychosocial conditions (e.g. social participation, job demand control), lifestyle (e.g. tobacco, alcohol, obesity, physical activity, diet), and access to essential services (e.g. education, health care) (Graham 2009). Especially for the association between material factors and health, there is widespread debate as to whether they reflect an effect of material factors on health (*social causation*) or the effect of health on material wealth (*health selection*) (Galama and van Kippersluis 2010; Martikainen et al. 2009). More specifically, it has been argued that the relative importance of the two pathways changes over the life course (Smith 2003). This may be because the sensitivity of health to a lack of material wealth and the degree to which health can influence economic resources depend on specific circumstances that change with age.

We study the question as to whether pathways from material wealth to health have more explanatory power than pathways from health to material wealth, and whether the relative importance of the two reciprocal effects changes over the life course. The causal direction between SES and health is part of a long-running discussion with several important normative and political implications regarding the reduction in health inequalities, which raises complex methodological questions concerning empirical analysis in a longitudinal life course framework. The life course perspective is crucial for understanding the interrelated processes of SES and health, and how observed health inequalities in old age develop. In the remainder of the introduction, we will elaborate on the open question of the relative importance of social causation and health selection, and why it may change with age.

### The reciprocal relationship between material wealth and health

Mechanisms that create health inequalities are manifold and have been discussed extensively in the literature (Case and Deaton 2005; Galama and van Kippersluis 2010; Hoffmann 2008). A thorough investigation reveals mechanisms where SES influences health and those where health affects SES. The first model assumes that material wealth influences health through, for example, the affordability of health care, environmental hazards, consumption, and the psychological burden of being poor. The second model involves health influencing material wealth, both via the career benefits associated with good health, and the financial costs incurred during illness. A third model to explain health inequalities is that (unknown) background factors influence both SES and health (indirect selection) (Goldman 2001). These factors may be genetic endowment, family background, or individual characteristics (genetic or acquired), such as height, personality, or preferences in behaviour and lifestyle.

There is disagreement concerning the relative importance of social causation versus health selection, due not only to different underlying ideas of the relation between social structure and presumably stable individual characteristics, but also to different research designs and methods, as well as to divergent concepts of causality. A central proposition of the health selection hypothesis is that social mobility is partly determined by health. While there are indications for a certain level of health-related social mobility at labour market entry (Smith 1999), the relationship between health and social mobility is fairly weak (Kröger 2015). Moreover, the chronology of social mobility at younger ages and increasing health problems at higher ages seems to contradict the proposition of the health selection hypothesis. Nevertheless, reverse causality from health to SES can bias the coefficients of conventional statistical models, and the direction of causality from SES to health should not be taken for granted.

Few epidemiological studies have examined the possibility of health selection (e.g. Chandola et al. 2003), and many authors believe that health selection is of little importance (e.g. Manor et al. 2003). The assessment of its relative importance also depends on the exact pathway under study. While there is agreement that education influences health (Gathmann et al. 2015; Lleras-Muney 2005), some scholars think that the influence of material resources on health is low, and that the influence of health on material status is the strongest overall causality in the relationship between SES and health (Galama and van Kippersluis 2010).

A recent systematic literature review of the relative importance of social causation versus health selection evaluated 34 out of 2952 reviewed studies from the past 20 years, qualitatively and in a quantitative, statistical meta-analysis (Kröger et al. 2015), concluding that there is no preference for one of the two directions. Twelve studies supported social causation, and 10 supported health selection—the other studies supported both mechanisms equally.

This study measures material wealth at three life course stages and covers large parts of the life course from childhood to old age, in order to estimate the relative explanatory power of social causation and health selection. The term material wealth is used in a broad sense to denote material living conditions in general, including income. We perform similar analyses with two comparable European data sets in order to explore whether a similar pattern of results can be found in two independent data sources. Our analysis does not allow us to study these differences in sufficient detail to interpret them as differences between countries or welfare systems. We use a long-term life course perspective and look at two broad age ranges, because the development of material wealth is accumulative (Dannefer 2003), and because specific mechanisms governing the interaction between material wealth and health may be relevant at specific stages of the life course. We are thus able to assess existing explanations for social causation and health selection at different stages of the life course. In particular, it has been suggested that health selection is relatively strong at labour market entry, when health influences occupation and income (Smith 1999), and in older working ages when many health problems start to become more prevalent (Oksanen and Virtanen 2012; Smith 2003). Unlike many existing studies, we do not aim to identify ‘local’ causal effects between a specific aspect of SES and a specific measure of health in a specific subgroup of the population. Instead, we use a long-term life course approach, use broad indicators of material wealth and health, and model their mutual influence simultaneously.

## Methods

### Data

We use the third wave (SHARELIFE, version 5.0.0) of the Survey of Health Aging and Retirement in Europe (SHARE) (Börsch-Supan 2016; Börsch-Supan et al. 2013), which covers the whole life course of respondents retrospectively. The second data source is the third wave of the English Longitudinal Study of Ageing (ELSA) (Marmot et al. 2017; Steptoe et al. 2012). In both surveys, persons aged 50 and older were asked retrospectively about changes in their material wealth and health since childhood. The data are representative for the population 50+ and their spouses living in households in the respective European countries (SHARE) and England (ELSA). From SHARE, we limit our analysis to ten countries (Austria, Belgium, Denmark, France, Germany, Italy, Netherlands, Spain, Sweden, and Switzerland), because for three SHARE countries information on wages was not comparable over the life course (Poland, Czech Republic) or contained too many missing values (Greece). We study persons aged 55–90 at the time of the interview in 2008/2009 (SHARE) and 2006/2007 (ELSA). Samples sizes are 18,734 (SHARE) and 6117 (ELSA). The average response rate across countries in SHARE Wave 1 is about 60% (ranging from about 40 to 80%). Details of participating countries are provided online (http://www.share-project.org/data-documentation/sample.html). SHARE added a refresher sample in Wave 2 to compensate for the loss of representativity due to follow-up attrition. The response rate in ELSA is 73%, and there was a refreshment sample in Wave 3. Details can be found in Steptoe et al. (Steptoe et al. 2012) for a description of the sample and the variables see Table 1.

**Table 1:** Samples description of SHARE and ELSA (variables, categories, distributions)

Latent construct	Variable	Category	SHARE		ELSA (England)	
			*N* = 18,734	(%)	*N* = 6117	(%)
	Country					
	West	Austria	945	5.0		
		Belgium	2584	13.8		
		France	2223	11.9		
		Germany	1762	9.4		
		Netherlands	2069	11.0		
		Switzerland	1157	6.2		
	South	Italy	2292	12.2		
		Spain	2035	10.9		
	North	Denmark	1806	9.6		
		Sweden	1861	9.9		
	Age in Wave 3 (SHARE 2008/2009, ELSA 2006/2007)	Mean	68.3		68.7	
		SD	8.9		9.0	
		Min	55		55	
		Max	90		90	
	Gender	Male	8598	45.9	2730	44.6
		Female	10,136	54.1	3387	55.4
C-MW (childhood material wealth)	Number of facilities	Mean	2.0		3.0	
		SD	1.8		1.4	
		Min	0.0		0.0	
		Max	5.0		5.0	
		Missing	129	0.7	271	4.4
	Rooms per capita	Mean	0.8		0.6	
		SD	0.4		0.2	
		Min	0		0.1	
		Max	10		3.8	
		Missing	326	1.7	291	4.8
C-H (childhood health)	Self-rated health	Poor	457	2.4	207	3.4
		Fair	1235	6.6	508	8.3
		Good	4777	25.5	1252	20.5
		Very good	5814	31.0	2070	33.8
		Excellent	6265	33.4	2021	33.0
		Missing	186	1.0	59	1.0
	Missed school	Yes	2166	11.6	1341	22.1
		No	16,441	87.8	4728	77.3
		Missing	127	0.7	48	0.8
	Hospitalized	Yes	1150	6.2	664	10.9
		No	17,489	93.4	5407	88.4
		Missing	95	0.5	46	0.8
A-MW (adult material wealth) Age 30–50	Owner of house or apartment	Yes	13,726	74.1	5054	83.5
		No	4792	25.6	996	16.3
		Missing	216	1.2	67	1.1
	Average wages (in SHARE corrected for purchasing power and inflation by purchasing power parities (PPP) relative to German € in 2006)	Mean	1301		1994	
		SD	868		1673	
		Min	8		83	
		Max	6126		12,826	
		Missing	9714	51.9	2182	35.7
A-H (adult health) Age 30–50	Percentage of years of non-illness	Mean	97.3		86.3	
		Min	0		0	
		Max	100		100	
	Percentage of years of non-poor health	Mean	97.5		NA	
		Min	0		NA	
		Max	100		NA	
O-MW (old age material wealth) Age 55–90	Household income in € (SHARE) and £ (ELSA)	Mean	35,290		15,396	
		SD	54,677		11,832	
		Min	0		0	
		Max	755,089		224,203	
		Missing	2132	11.4	102	1.7
	Household wealth in € (SHARE) and £ (ELSA)	Mean	161,356		66,842	
		SD	222,142		162,377	
		Min	− 784,644		− 81,495	
		Max	7153,102		3,631,500	
		Missing	618	3.3	103	1.7
O-H (old age health) Age 55–90	Self-rated health	Poor	2336	12.5	32	0.5
		Fair	5116	27.3	199	3.3
		Good	6895	36.8	1127	18.4
		Very good	2886	15.4	2088	34.1
		Excellent	1424	7.6	1181	19.3
		Missing	77	0.4	1490	24.4
	Grip strength	Mean	33.6		29.6	
		SD	12.2		11.4	
		Min	1		0.0	
		Max	85		70	
		Missing	1576	8.4	1502	24.6
Alternative measures	Lung function (spirometer)	Mean	3.6		3.8	
		SD	1.7		1.4	
		Min	0.3		0.4	
		Max	10.0		9.5	
		Missing	3612	19.3	1772	29.0
	Number of limitations in activities of daily living (ADL)	Mean	0.2		0.3	
		SD	0.7		0.9	
		Min	0.0		0.0	
		Max	6.0		6.0	
		Missing	2585	13.8	160	2.6

To measure limitations in ADL, respondents are asked whether they have any difficulty with (1) dressing, including putting on shoes and socks, (2) walking across a room, (3) bathing or showering, (4) eating, such as cutting up your food, (5) getting in or out of bed, and (6) using the toilet, including getting up or down. They are asked to exclude any difficulties that they expect to last less than 3 months

### Measures

In the operationalization of our concepts, we divide the life course into three periods: childhood (age 0–15), adulthood (age 30–50), and old age (age 55–90). For childhood, we use indicators available in SHARE and ELSA that refer to childhood in general or to individuals at age ten; for adulthood, we use retrospective information and calculate averages for the age range 30–50; old age is represented by prospective information at the time of the interview, which ranges from age 55 to 90.

The starting age of the oldest group is set at age 55, because we use the third wave of SHARE that was representative of the population aged 50+ in its first wave 6 years earlier. We use heterogeneous measurement between age groups; it is the only way to combine retrospective and prospective survey data and allows us to measure health and material wealth at very different ages.

We use two indicators for material wealth in childhood that are rough indicators for the general standard of living, but easy for interviewees to remember: the number of rooms per person and a summary index of features of the household (cold water, hot water, toilet, bath, heating). For adulthood, we use the two indicators homeownership and estimates of average monthly wages between age 30 and 50, corrected for purchasing power and inflation by purchasing power parities (PPP) relative to the level of Germany in the year 2006 (Weiss 2012).

Respondents specified the beginning and the end of episodes in which they were home owners or rented, and we used the mode of this variable. Likewise, respondents specified job spells and reported their wages, which we averaged over the adult age range, taking into account the lengths of the spells. These reports also take into account periods of unemployment and promotions or wage changes within the same job. Wages represent monetary wealth, while home ownership provides information about the general stability of material circumstances, although it does not necessarily indicate the standard of living. In higher age groups, we measure material wealth with the net-equivalent household income at the time of the interview. Alternatively, we measure it with household net wealth per capita (property, cars, company shares, and liquid funds, minus debts). We analyse income and wealth separately in old age to establish whether they produce different results. This could be due not only to the different effects on health suggested in the literature (Avendano and Glymour 2008), but also to their different degree of responsiveness to health; wealth is even more stable than (pension) income because it has been acquired over the whole life course. Results using wealth are in the Online Resources.

Health in childhood is measured retrospectively by three indicators: self-assessed health in five categories, whether school was missed because of health for 1 month or more, and whether 1 month or more was spent in hospital. At ages 30–50, our health measure for SHARE is based on two indicators reflecting how many years (as a share of the years between age 30 and 50) individuals reported being either in bad health in general, or suffering from an acute or chronic illness. For ELSA, only the first of these indicators was available, used as a manifest variable. In old age, health is measured with the indicators current self-rated health (SRH) and grip strength. SRH is considered a good health measure and predictor for mortality. It measures health not only as the absence of disease, but comprehensively (Idler and Benyamini 1997). Grip strength is an objective measure and has been shown to be related to income, and even more so to wealth (Mohd Hairi et al. 2010). In sensitivity analyses, we use an index of six limitations in activities in daily living (ADL) and a measurement of lung function (a spirometer measurement of how much air respondents can exhale, which has been shown to be related to general health) (Sabia et al. 2010). Table 1 shows that the distributions of SRH differ strongly between SHARE and ELSA. Since our models exploit the covariation of variables within one data set, this does not bias our results. We do not include further control variables because our aim is to estimate the total effects between material wealth and health, and we expect other variables either to mediate these effects (e.g. health behaviour) or to affect health through income (e.g. occupation).

### Analysis

We chose a model-based approach to study the interplay between material factors and health across the life course. The advantage of a model-based approach, compared to design-based approaches such as quasi-experiments, is the potential for simultaneously modelling two related processes (social causation and health selection) in which the outcome of one process is the predictor of the other.

**Fig. 1:**
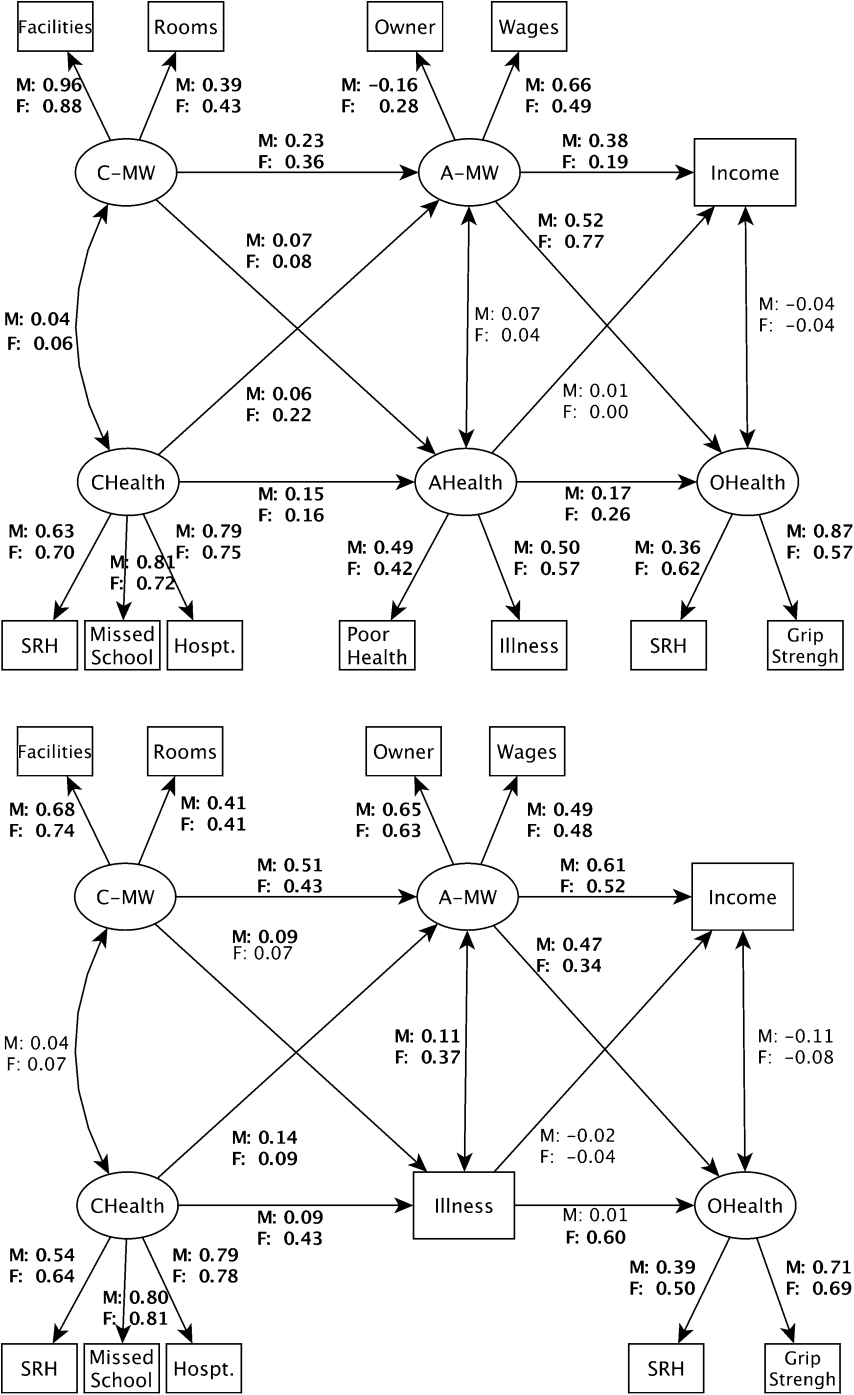
Structural equation model for reciprocal relationships between material wealth and health over the life course, with standardized coefficients, factor loadings, and simultaneous correlations between health and material wealth, for SHARE (upper panel) and ELSA (lower panel). *Notes* (a) observed variables are shown as boxes and latent variables as ellipses; uni-directed arrows are path coefficients or factor loading from the measurement models, bi-directed arrows are correlations; (b) SHARE countries in the upper panel are Austria, Germany, Netherlands, France, Switzerland, Belgium, Sweden, Denmark, Spain, Italy; (c) *C* childhood (0–15), *A* adulthood (30–50), *O* old age (55–90), *MW* material wealth, *M/F* male/female; (d) bold numbers are statistically significant (*p* < 0.05)

We estimate the parameters of a structural equation model (Bollen 1989; Pakpahan et al. 2015) that includes social causation and health selection in different stages of the life course. Our model is represented by a cross-lagged panel design (Fig. 1). We model material wealth and health at three different ages as latent variables with measurement models, except for adult health in ELSA (which only offers one observed variable) and material wealth in old age, where we explicitly compare the results for the observed variables income and wealth. The parameters are estimated using mean and variance-adjusted weighted least squares (WLSMV) (Finney and DiStefano 2006). We present standardized coefficients in a uniform value range of − 1 to 1, making them comparable across paths and models. Our model estimates the correlation between material wealth and health in childhood that can be jointly influenced by common unobserved background factors, e.g. genetic factors or unobserved characteristics of the family. Consequently, we address the common background factors mentioned above to the extent that such factors create a correlation between health and wealth in childhood. The path parameters can be divided in two groups: first, the autoregressive parameters showing the effect of wealth at *t*_1_ on wealth at *t*_2_ (and the same for health); second, the cross-lagged parameters showing how wealth at *t*_1_ influences health at *t*_2_ (social causation) or health at *t*_1_ influences wealth at *t*_2_ (health selection). In the SHARE analysis, we use country dummies to control for unobserved national differences. All models are calculated separately for men and women, and age at interview, in 5-year categories reflecting the birth cohort, is also controlled for. Data preparation is performed in Stata 14.1 and analyses in Mplus 7.4 (Muthen and Muthen 2015).

## Results

**Fig. 2:**
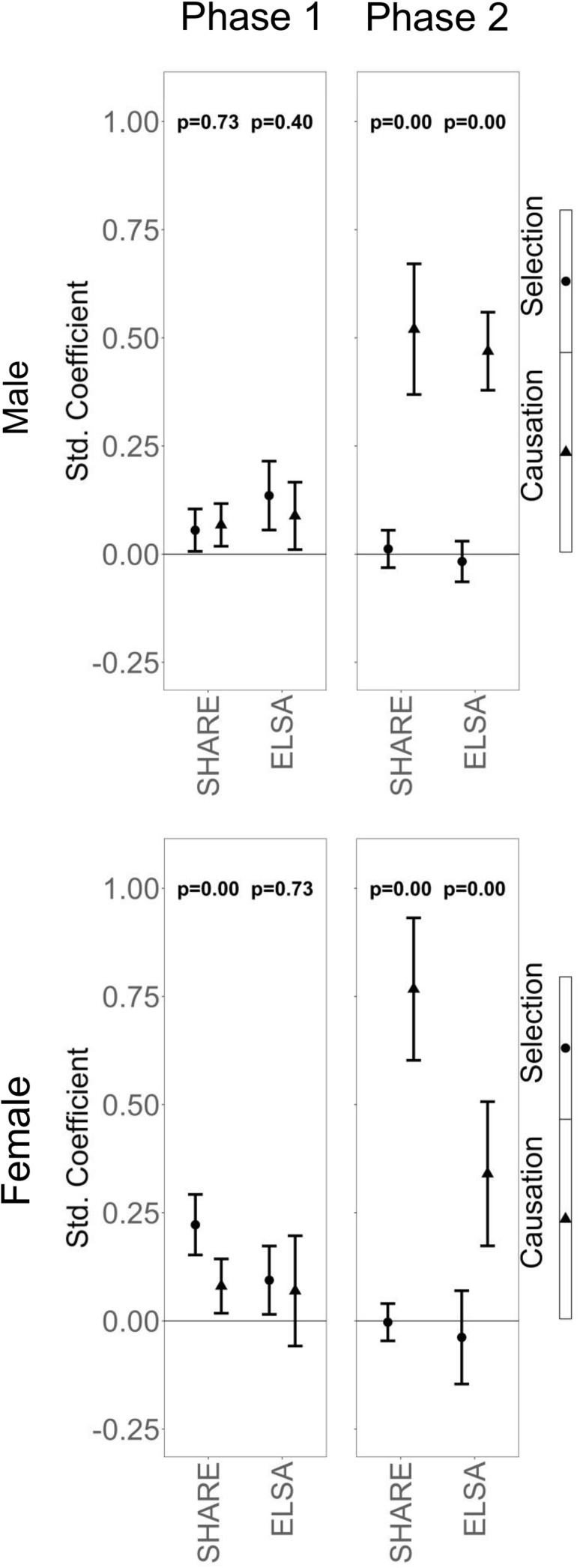
Relative explanatory power of social causation and health selection, by life course phase and gender. *Notes* (a) Phase 1 = transition from childhood to adulthood; Phase 2 = transition from adulthood to old age; (b) the confidence intervals show whether an estimate is different from zero (*p* < 0.05), while the *p* values in the graph are from a direct Wald test for difference between the standardized coefficients for social causation and health selection

Results from the structural equation models are shown in Fig. 1 (as a graphical illustration of the model and overview with results for SHARE and ELSA), in Table 2 (all coefficients, standard errors, and goodness-of-fit measures), and in Fig. 2 (only results that are relevant for our main question, the comparison between social causation and health selection). Goodness-of-fit statistics for the measurement models are provided in Online Table 1.

**Table 2:** Results from structural equation models on the relationship between material wealth and health over the life course

	Parameter		Male		Female	
			SHARE	ELSA	SHARE	ELSA
Correlation	C-MW ↔ CHEALTH	Coef.	0.04	0.04	**0.06**	**0.07**
		SE	0.02	0.04	0.02	0.04
	A-MW ↔ AHEALTH	Coef.	0.07	**0.11**	0.04	**0.37**
		SE	0.07	0.04	0.08	0.11
	O-MW ↔ OHEALTH	Coef.	− 0.04	− 0.11	− 0.04	− 0.08
		SE	0.03	0.07	0.07	0.13
*Phase 1*						
Autoregression	C-MW → A-MW	Coef.	**0.23**	**0.51**	**0.36**	**0.43**
		SE	0.03	0.04	0.04	0.04
	CHEALTH → AHEALTH	Coef.	**0.15**	**0.09**	**0.16**	**0.43**
		SE	0.02	0.03	0.02	0.05
Causation	C-MW → AHEALTH	Coef.	**0.07**	**0.09**	**0.08**	0.07
		SE	0.03	0.04	0.03	0.06
Selection	CHEALTH → A-MW	Coef.	**0.06**	**0.14**	**0.22**	**0.09**
		SE	0.02	0.04	0.04	0.04
*Phase 2*						
Autoregression	A-MW → O-MW	Coef.	**0.38**	**0.61**	**0.19**	**0.52**
		SE	0.08	0.03	0.05	0.04
	AHEALTH → OHEALTH	Coef.	**0.12**	0.01	**0.26**	**0.60**
		SE	0.02	0.03	0.04	0.08
Causation	A-MW → OHEALTH	Coef.	**0.52**	**0.47**	**0.77**	**0.34**
		SE	0.08	0.05	0.08	0.08
Selection	AHEALTH → O-MW	Coef.	0.01	− 0.02	0.00	− 0.04
		SE	0.02	0.02	0.02	0.06
		Chi2	2105	458	1393	542
		*p* Value	0	0	0	0
		CFI	0.782	0.882	0.877	0.879
		TLI	0.610	0.805	0.781	0.800
		RMSEA	0.038	0.062	0.029	0.061
	RMSEA 90% confidence interval:	Lower	0.036	0.057	0.028	0.056
		Upper	0.039	0.067	0.031	0.066

(a) Standardized regression coefficients; *SE* standard errors, *C* childhood, *A* adulthood (30–50), *O* old age (55–90), *MW* material wealth; Phase 1 = transition from childhood to adulthood; Phase 2 = transition from adulthood to old age; (b) for interpretation of the coefficients, e.g. 0.5 means that one standard deviation change in the independent variable results in 0.5 standard deviation change in the dependent variable; (c) statistically significant coefficients (*p* < 0.05) are printed in bold; (d) the total results for SHARE are weighted to account for unequal probability in the sampling process and to represent the different sizes of the population in the countries in Europe

As expected, the correlation between childhood material wealth (C-MW) and childhood health (C-H) is positive. However, the coefficient is only statistically significant in SHARE. All but one of the autoregressive coefficients for MW and health in both phases of the life course are statistically significant, and they range from 0.01, which means almost no path dependency from adult to old age health (among men in ELSA), to 0.61 in the same group, which means that one standard deviation increase in A-MW is associated with a 0.61 standard deviation increase in O-MW.

The effects from C-H to A-MW (health selection) are somewhat stronger than from C-MW to A-H (social causation), but the total cross-lagged effects from childhood to adulthood suggest similar explanatory power for social causation and health selection among men. This is also displayed in Fig. 1. Among women in SHARE, health selection seems to be somewhat stronger than social causation, with 0.22 versus 0.08. Altogether, out of eight coefficients that concern reciprocal effects in the first phase, seven are statistically significant, ranging between 0.06 (health selection for men in SHARE) and 0.22 (health selection for women in SHARE), classifiable as relatively small effects that do not show systematic differences between data sets and gender. Summarizing the transition from childhood to adulthood, both reciprocal effects are of equally small importance, with a tendency towards stronger health selection among women.

For the transition to old age, our model shows that all coefficients for social causation are positive and statistically significant, and range between 0.34 (women in ELSA) and 0.77 (women in SHARE), showing a strong predictive power of adult MW on health in old age. The coefficients for health selection are much smaller than for social causation, around zero, and none of them is statistically significant.

The goodness-of-fit indicators show variable results: the Chi-square is always highly significant and the root-mean-square error of approximation (RMSEA) shows good fit for SHARE and acceptable fit for ELSA, the latter with a maximum value of 0.062 (CI 90% 0.057–0.067). The comparative fit index (CFI) and the Tucker–Lewis index are borderline, with ranges between 0.782 and 0.882 and between 0.610 and 0.805, respectively. However, we think that the lower values of CFI and TLI are acceptable, because the goal of the study is the comparison of the social causation and health selection pathways, and not a best possible model of all interrelationships of material factors and health throughout the life course.

For a more direct comparison of the two reciprocal effects, Fig. 2 shows only the related coefficients for SHARE and ELSA, by life course stages and gender. For men in the transition from childhood to adulthood (Phase 1), both pathways have the same explanatory power and they are both statistically significant (indicated by the box-plots), but the difference between them is not statistically significant (indicated by the *p* values in the graph). Health selection is unimportant during the transition to old age (Phase 2), but social causation is stronger than before and significantly stronger than health selection. Among women, we see similar results in the second phase, but in the first phase, health selection seems to be stronger than social causation in SHARE, which means a reversal of the relative importance of social causation and health selection during the life course among women. The findings are the same for SHARE and ELSA. The same results as in Fig. 2, but with wealth as a measure for material wealth in old age, can be found in Online Fig. 1. Using wealth, the main findings stay the same, except that social causation and health selection in Phase 1 are equally important for women. In other words, the only exception to the overall pattern that we found in the results based on income disappears when we use wealth. This highlights the fact that alternative measures influence the results, but the overall pattern is robust with different indicators for material wealth. The results are also similar between models where age at interview is controlled for and those where it is not. This suggests neither the age of the respondent, the time elapsed between the period discussed in the interview and its actually taking place, nor the birth cohort matter for the relative importance of social causation versus health selection.

## Discussion

This study showed in a comprehensive life course perspective that, firstly, material factors and health substantially depend on their prior status and, secondly, that in the transition from childhood to adulthood, the health selection path is as important as the social causation path, while in the transition from adulthood to old age, social causation was much more important than health selection. Health selection was marginally more important than social causation in the transition from childhood to adulthood among women in SHARE. We discuss this exception below, because it is related to our research question. Beyond that, we did not find any systematic differences between SHARE and ELSA, nor between men and women, that deviate from our established general pattern. Small differences between data sets and gender do not warrant a substantial interpretation because of the limited statistical power of our study.

The near-equal significance of social causation and health selection corroborates previous findings on relatively high social mobility at labour market entry, when health influences occupation, which influences income (Smith 1999). Stronger health selection among women than among men in SHARE is surprising. We can only conclude that health selection on the labour market, i.e. health discrimination and self-selection related to lower female labour force participation rates, is at least as strong among women as among men and speculate that health selection on the marriage market and other mechanisms might also be involved. It is noteworthy that this reversed pattern in Phase 1 for women in SHARE does not appear in the analysis based on wealth. Thus, we think that a substantial interpretation of this finding in terms of real differences between gender or data sources is not warranted.

The high percentages of good health in adulthood (means are between 86 and 98%) contribute to small coefficients for social causation between childhood and adulthood. We concede that a different measure for adult health with more variance could have resulted in more social causation, but on the other hand, the low prevalence of bad health in adulthood is not unrealistic and implies that differences in childhood material wealth did not influence health in adulthood to a large extent.

Much stronger evidence for social causation (compared to health selection) in Phase 2 contradicts previous evidence that health selection is especially important in older working ages, where many health problems start to become more prevalent (Oksanen and Virtanen 2012; Smith 2003). Our findings suggest instead that in the welfare systems under investigation, health problems at older working ages do not lead to significant losses in material wealth; the evidence may also be explained by the high percentage of retired persons in the age range 55–90 (51.5% for men and 35.3% for women), whose material wealth can no longer easily be influenced by health. To test whether our results for the wide age group 55–90 differ between people who are still working (where health selection may be important) and retired people (where health selection should occur much less), we separated these two groups, keeping age as a control variable. The results (not shown) do indeed indicate that health selection is slightly higher among occupied people.

Our results show that the question of social causation versus health selection needs to be discussed within a life course framework, as its answer depends on the life stage studied. Several earlier studies investigate the relative importance of social causation and health selection, but do not examine interactions with age, which should be a topic for future research. Studies with similar age groups, indicators, and methods show comparable results. Mulatu and Schooler (2002) use prospective data for the age range 41–88, corresponding only to our second life phase, and show that social causation is slightly more important than health selection. Warren (2009) studies the age range 18–65, showing that childhood health has no effect on educational achievement. Interestingly, he finds no evidence for health selection; although he establishes no correlation between childhood health and education, his main test for selection is ultimately for the age group 54–65. In our age range 55–90, we do not find health selection either. Warren also finds similar results for three different health measures. Aittomäki et al. (2012) use Finnish register data (age range 17–66) and show social causation to be slightly more important than health selection. Other authors claim that evidence on the relative importance of social causation and health selection will always be contingent on the social context, the method, and the indicators used (Huurre et al. 2005). These indicators cannot be assessed on a simple gradient of more or less validity; particular dimensions of SES are probably related via specific mechanisms to certain aspects of health. We agree and see it as a challenge for future research to address this theoretical and empirical complexity. For example, future research should check our results with other indicators for SES. If education or occupation is used instead, health selection might play a smaller role, because these indicators are less prone to health selection (Galama and van Kippersluis 2010; Martikainen et al. 2009).

In the comparison of social causation and health selection, the third model of indirect selection also needs to be discussed. It assumes that SES and health are determined by common background factors, such as innate or acquired cognitive or physical characteristics (O’Rand et al. 1999), which can lead to the development of specific personalities or lifestyle preferences (Fuchs 1982). It is difficult to empirically measure such common background factors and related mechanisms. The problem with any existing variable (IQ, non-cognitive traits, school performance, birth weight, height, etc.) is that it may already depend on prior SES (of the individuals’ parents). We understand our finding that health and wealth in childhood are only weakly correlated as implying that indirect selection is not of major importance, because common background factors would presumably create such correlation. However, it is theoretically possible that these factors exist, with their effects only materializing later in life; here, too, we see very little correlation between health and wealth net of previous cross-lagged effects that are taken into account when the model estimates the correlations. Previous studies that use a different design and a different definition of indirect selection come to contrary conclusions: Foverskov and Holm, for example, (2016) begin observing the relationship between SES and health at age 30, find little or no mutual effects, and conclude that health inequalities can be explained by indirect selection, which they define as everything before age 30. An advantage of our study is that we start measuring SES and health as early as possible in the life course, thus attributing as much as possible of their interrelation to either social causation or health selection, instead of using indirect selection as a black box or residual model that absorbs all interactions before observations began.

### Strengths and limitations

Our study combines a number of innovative strengths; first, we start early in the life course by measuring the very beginning of the development of health and SES, gradually proceeding to old age. This is crucial for disentangling related processes (Heckman 1981). Second, we include several indicators that are important for a valid measurement of material wealth and health, and we combine these indicators into measurement models for latent variables, which reduce measurement error. The influence of measurement error on results and conclusions in a cross-lagged panel design has been shown in previous research (Kröger et al. 2016). Third, we use structural equation models that can simultaneously model two pathways, also taking into account indirect selection, at least to the extent to which it creates a correlation between health and wealth. Fourth, we study two high quality data sets, and we compare two age groups in order to address age-specific mechanisms that might determine the relative importance of social causation and health selection.

Some limitations to our approach remain. The fact that our data cover a long time span comes at the cost of using retrospective data that, in principle, might be affected by recall bias (Smith and Thomas 2003). Measurement error in childhood variables can bias the association between childhood and later life outcomes. However, several studies have shown that the retrospective measurement of health and SES, including the SHARE data, is relatively valid (Garrouste and Paccagnella 2011; Mazzonna and Havari 2011). The disadvantages of these data need to be weighed against the fact that it enables the study of longer periods than in previous research based on prospective data (Stowasser et al. 2011). Latent variables reduce measurement error by using several indicators for a latent concept, where more reliable (objective) indicators may complement less reliable ones. Still, the choice of variables to measure complex concepts such as health and material wealth was limited, and the sensitivity of the results to the choice of measures remains an important issue. We include two sensitivity analyses where alternative measures are used: first, an analysis where CH–H is measured by SRH only, instead of SRH, missed school, and hospitalization, and where O–H is measured by SRH only, instead of SRH and grip strength, and second, an analysis where O–H is measured by ADL and lung function. With one exception in each of these sensitivity analyses (social causation and health selection are not similar in Phase 1, and they are not statistically different in Phase 2), which we attribute to overall variability in a complex model, the sensitivity analyses confirm the overall pattern of results (see Online Figs. 2 and 3). However, our conclusions are still tentative, because we cannot claim that all other possible measures of health and material wealth would also yield the same result.

As mentioned in the data section, the response rate of SHARE is slightly lower than for ELSA. Assuming that non-response is not random, this might imply an underrepresentation of poor and unhealthy people. A similar problem exists for missing values that might not be missing at random. The highest percentage of missing values in our survey data is for the variable ‘wages’ (51.9%). We performed a sensitivity analysis, excluding cases with missing wages, and found that, besides minor changes, this does not change the main results (see Online Fig. 4). Many of these missing wages are from individuals who are primarily taking care of home and family. We thus conducted a second sensitivity analysis excluding 1241 women and 51 men from the SHARE sample who reported, for at least 75% of the years between ages 30 and 50, to have mainly worked in the household. This, too, did not change the results (see Online Fig. 5).

We explored whether our findings are sensitive to the inclusion of direct paths from childhood to old age, and we found that these paths were not statistically significant and did not change the relative magnitude of social causation and health selection (results not shown). This confirms existing evidence that most of the effects of childhood on old age (‘the long arm of childhood’) are moderated by SES and health in adulthood (Pakpahan et al. 2017).

Our analysis does not take mortality into account, which is a good health indicator, but logically cannot be used to predict changes in wealth in a model. We only study the surviving population, which might be selected, but we assume that, while selective mortality decreases health inequality in the surviving population, e.g. by poor and unhealthy people dying first, it does not systematically bias the comparison between social causation and health selection. This would only be the case if people who are poor and unhealthy, and thus underrepresented in the selected sample, were represented more in one of the two causal pathways than in the other.

Last, our latent variable modelling approach does not enable the identification of group-specific trajectories of SES and health (as in latent class analysis), which would be an interesting complementary analysis for future research.

### Conclusion and implications

Both social causation and health selection play a part in the creation of health inequalities over the life course. In the second part of the life course, social causation is more important than health selection. This study contributes to the debate on the two different mechanisms by assessing the relative contribution of each mechanism to health inequality. Their relative importance has implications for the normative assessment of health inequalities: The liberal or meritocratic claim that health selection is less unfair than social causation assumes that a significant amount of individual differences in health are caused by biology alone, unrelated to SES. Instead, we would propose the welfare-state perspective that social causation and health selection are equally unfair, because both ill health as a consequence of poverty and poverty resulting from ill health indicate a dysfunction of the social security system that should, in principle, counteract both pathways. If both mechanisms contribute to health inequalities, both could also be used as entry points for social policy to reduce health inequalities. Our second contribution to the knowledge base on health inequalities is that the relative contribution of social causation and health selection is very different in different stages of the life course. This improves our understanding of the mechanisms behind health inequalities, lending more plausibility to health-related social mobility in younger ages, and less plausibility to substantial health selection in older working ages. Social causation seems to accumulate and increase with age. However, finer age differentiation is needed in future studies to confirm the relative importance of concrete mechanisms. This age differentiation would also have the potential to inform policy on which mechanisms to address at which stages in the life course, in order to more effectively reduce health inequalities.

## Electronic supplementary material

Below is the link to the electronic supplementary material.

Supplementary material 1 (EPS 245 kb)

Supplementary material 2 (EPS 246 kb)

Supplementary material 3 (EPS 260 kb)

Supplementary material 4 (EPS 246 kb)

Supplementary material 5 (EPS 245 kb)

Supplementary material 6 (DOCX 20 kb)
