# Supplementary material for: The reciprocal relationship between material factors and health in the life course: evidence from SHARE and ELSA
Source: Eur J Ageing. 2018 Feb 19;15(4):379–91. doi: 10.1007/s10433-018-0458-3 (PMC6250643; doi:10.1007/s10433-018-0458-3)
Supplement: Supplementary file 6 — Supplementary material 6 (DOCX 20 kb) [file 10433_2018_458_MOESM6_ESM.docx]

**Online Table 1.** Goodness of fit indices of five measurement models

| **SHARE** | **Male** | | | | |  |  | **Female** | | | | |
| --- | --- | --- | --- | --- | --- | --- | --- | --- | --- | --- | --- | --- |
|  | C-SES | C-H | A-SES | A-H | O-H |  |  | C-SES | C-H | A-SES | A-H | O-H |
| CFI | 1.000 | 1.000 | 1.000 | 1.000 | 1.000 |  | CFI | 1.000 | 1.000 | 1.000 | 1.000 | 1.000 |
| TLI | 1.000 | 1.000 | 1.000 | 1.000 | 1.000 |  | TLI | 1.000 | 1.000 | 1.000 | 1.000 | 1.000 |
| RMSEA | 0.000 | 0.000 | 0.000 | 0.000 | 0.000 |  | RMSEA | 0.000 | 0.000 | 0.000 | 0.000 | 0.000 |
| WRMR | 0.000 | 0.000 | 0.028 | NA | 0.000 |  | WRMR | 0.000 | 0.018 | 0.033 | NA | 0.000 |
| SRMR | NA | NA | NA | 0.000 | NA |  | SRMR | NA | NA | NA | 0.000 | NA |
|  |  |  |  |  |  |  |  |  |  |  |  |  |
| **ELSA** | **Male** | | | | |  |  | **Female** | | | | |
|  | C-SES | C-H | A-SES |  | O-H |  |  | C-SES | C-H | A-SES |  | O-H |
| CFI | 1.000 | 1.000 | 1.000 |  | 1.000 |  | CFI | 1.000 | 1.000 | 1.000 |  | 1.000 |
| TLI | 1.000 | 1.000 | 1.000 |  | 1.000 |  | TLI | 1.000 | 1.000 | 1.000 |  | 1.000 |
| RMSEA | 0.000 | 0.000 | 0.000 |  | 0.000 |  | RMSEA | 0.000 | 0.000 | 0.000 |  | 0.000 |
| WRMR | 0.000 | 0.005 | 0.011 |  | 0.000 |  | WRMR | 0.000 | 0.000 | 0.019 |  | 0.000 |
| SRMR | NA | NA | NA |  | NA |  | SRMR | NA | NA | NA |  | NA |

Note: A-H is based on MLR estimation instead of WLSMV, because all indicators are continuous variables. This leads to different availability of fit indices. In ELSA, no measurement model is used for A-H.
